# A simple method for determining dosimetric leaf gap with cross-field dose width for rounded leaf-end multileaf collimator systems

**DOI:** 10.1186/s13014-018-1164-1

**Published:** 2018-11-13

**Authors:** Chih-Yuan Lin, An-Cheng Shiau, Jin-Huei Ji, Chia-Jung Lee, Ti-Hao Wang, Shu-Hui Hsu, Ji-An Liang

**Affiliations:** 1 Department of Radiation Oncology, China Medical University Hospital, No.2, Yude Rd., North Dist, Taichung City, 404 Taiwan; 2 Department of Biomedical Imaging and Radiological Sciences, National Yang-Ming University, Taipei, 112 Taiwan; 3 Department of Biomedical Imaging and Radiological Science, China Medical University, Taichung, 404 Taiwan; 4 Department of Medicine, China Medical University, Taichung, 404 Taiwan; 5 Department of Radiation Oncology, Montefiore Medical Center, Bronx, NY USA

**Keywords:** Dosimetric leaf gap, MLC, Treatment planning systems, GAFCHROMIC film

## Abstract

**Purpose:**

The dosimetric leaf gap (DLG) and multileaf collimator (MLC) transmission are two important systematic parameters used to model the rounded MLC leaf ends effect when commissioning an Eclipse treatment planning system (TPS). Determining the optimal DLG is a time consuming process. This study develops a simple and reliable method for determining the DLG using the cross-field dose width.

**Methods and materials:**

A Varian TrueBeam linac with 6 MV, 10 MV, 6 MV flattening filter free (FFF) and 10 MV FFF photon beams and equipped with the 120 Millennium MLC and the Eclipse™ TPS was used in this study. Integral sliding fields and static slit MLC field doses with different gap widths were measured with an ionization chamber and GAFCHROMIC EBT3 films, respectively. Measurements were performed for different beam energies and at depths of 5 and 10 cm. DLGs were derived from a linear extrapolation to zero dose and intercepting at the gap width axis. In the ion chamber measurements method, the average MLC leaf transmission to the gap reading for each gap (*R*_*gT*_) were calculated with nominal and cross-field dose widths, respectively. The cross-field dose widths were determined according to the dose profile measured with EBT3 films. Additionally, the optimal DLG values were determined using plan dose measurements, as the value that produced the closest agreement between the planned and measured doses. DLGs derived from the nominal and cross-field dose width, the film measurements, and the optimal process, were obtained and compared.

**Results:**

The DLG values are insensitive to the variations in depth (within 0.07 mm). DLGs derived from nominal gap widths showed a significantly lower values (with difference about 0.5 mm) than that from cross-field dose widths and from film measurements and from plan optimal values. The method in deriving DLGs by correcting the nominal gap widths to the cross-field dose widths has shown good agreements to the plan optimal values (with difference within 0.21 mm).

**Conclusions:**

The DLG values derived from the cross-field dose width method were consistent with the values derived from film measurements and from the plan optimal process. A simple and reliable method to determine DLG for rounded leaf-end MLC systems was established. This method provides a referable DLG value required during TPS commissioning.

## Background

The dynamic multileaf collimator (dMLC) has been widely used to achieve beam-intensity modulation for a high conformity modern radiotherapy dose distribution. The configurations of most MLC systems have rounded leaf tips with rectilinear leaf motion. Additional x-ray transmission through the leaf ends causes a discrepancy between the dosimetric and geometric field widths, and an offset from the geometric leaf position should be applied [1]. The relationship between the MLC design and radiation dosimetry, and the MLC leaf position specifications have been interpreted clearly by Philip Vial et al. [2]. The radiation field offset (RFO) accounts for a single leaf offset while the dosimetric leaf gap (DLG) accounts for the opposing leaves offset and MLC transmission. There are two important systematic parameters in the treatment planning system (TPS) that accurately model the dosimetric distribution in a dynamic MLC plan. The DLG is a systemic change to the MLC leaf position, and the variations can cause significant dosimetric deviation, especially for more complex MLC leaf motion. To maintain adequate dose accuracy in clinical applications for dynamic MLC plans like Intensity-Modulated Radiotherapy (IMRT) or Volumetric-Modulated Arc Therapy (VMAT), systematic errors in the DLG need to be minimized. Oliver et al [3] concluded that MLC open and close errors should be within 0.6 mm to keep the dose variation in the target (PTV70) coverage (PTV70) within 2%. Similar results have also been reported. [4–6]

The DLG is affected by the x-ray transmission through the rounded leaf ends, and therefore the value would depend on the beam quality and MLC type. DLG values are usually determined for each beam energy during commissioning. To determine the DLG values, the integrating cross-field dose technique [5] and sweeping gap technique [7] are widely used. For a Varian system, the sweeping gap technique as described in Varian Medical Systems’ documentation provides the most convenient method to derive the DLG [7]. However, several studies reported that the DLG value measured with this method had a significant difference from the value determined by optimizing the DLG value such that the differences between TPS calculations and delivered doses were minimal for clinical plans [8–13]. This discrepancy, without a referable baseline, makes the optimizing DLG value process time consuming. Additionally, from these reports, a trend has been noticed that the DLG values measured according to the manufacturer’s guidelines were usually smaller than the values derived using the optimized clinical plan. As we reevaluated the calculation equations used in the vender-provided document, we found that the possible reason for this discrepancy might be the calculation equations in the manufacturer’s method are based on the geometrical (nominal) relations of the gap size, especially for the calculation of average MLC leaf transmission to the gap reading for each gap (*R*_*gT*_). However, DLG and MLC leaf transmission in these equations are dosimetric related, the sliding field gap width in the equation to calculate *R*_*gT*_ should take the dosimetric distribution into account.

By using the dosimetric-based concept, the cross-field dose width was used in this study, and a simple and reliable method for determining the DLG was developed. Additionally, the DLG values derived in this method were compared with those values acquired by the integrating cross-field dose measured by film and the values optimized by using the clinical plans.

## Materials and methods

### Linac, MLC, TPS, measurement devices and setup

All measurements were conducted on a Varian TrueBeam (Varian Medical Systems, Palo Alto, CA) machine equipped with a Millennium 120 leaf MLC. Beam energies of 6, 10 MV for flattened and flattening-filter-free (FFF) beams were used. All dose calculations were performed using an Analytical Anisotropic Algorithm (AAA, v13.6.23) in an Eclipse TPS (Varian Medical Systems Inc., Palo Alto, CA). Plastic solid water phantom (PlasticWater™, CIRS), 0.6 cc Farmer ionization chamber (PTW TN30013, Freiburg, Germany) and GAFCHROMIC EBT3 film (International Specialty Products, Wayne, NJ, currently Ashland, KY) were used for dose measurements. The ion chamber and EBT3 films perpendicular to the beam axis were placed at the isocenter in the solid phantom at depths of 5 and 10 cm.

### Deriving DLG with sweeping gap technique using ion chamber

The initial DLG values were calculated following the methodology described by LoSasso et al. [5] but using an ion chamber and the Varian supplied DICOM files for the sweeping gap measurements. The integral ionizations were measured at depths of 5 and 10 cm for nominal gap widths of 2, 4, 6, 10, 14, 16 and 20 mm, respectively. The sweeping gap moved from − 60 mm to 60 mm with a constant speed.

To actually accumulate the ionization contributed only by the sweeping gap field, the MLC transmission reading during the slit movement should be subtracted, as the chamber was totally shielded by the leaves. The *R*_*gT*_ and the corrected gap reading (*Rg*^′^) for each gap (*g*) are defined by manufacturer’s guideline as:

1 $$ {R}_{gT}={R}_T\bullet \left[1-\frac{g\ (mm)}{120(mm)}\right], $$

2 $$ {R}_{g^{\prime }}={R}_g-{R}_{gT}, $$

where the *R*_*T*_ is the average MLC leaf transmission accounted for MLC bank A and B, the *g(mm)* is the nominal gap width, the 120 (mm) is the sweeping gap movement range and *R*_*g*_ is the initial sweeping gap field reading. A linear regression analysis was applied with the *Rg*^′^ plotted against the nominal gap width, and the absolute intercept value of the fitted function provides a DLG result.

The cross-field dose size would account for the whole range of dose distribution. To integrate the net dose contribution from a sweeping gap field, MLC transmission (*RgT*) subtraction from *R*_*g*_ in eq. (2) should revise the value of *g*(mm) in eq. (1) from the nominal gap width to the cross-field dose width. The cross-field dose widths (*g*_*D*_) were determined using film measurements. Equation (1) calculated with nominal gap width (*g*_*N*_) and cross-field dose width (*g*_*D*_) were performed, respectively.

### Deriving DLG with integrating cross-field dose technique using EBT3 film

The film measurement settings were the same as the measurements for the ion chamber, but a fixed field was used instead of the sweeping field. The jaw field was set to 28 × 40 cm^2^ for all MLC fields. The nominal MLC gap sizes of 2, 4, 6, 10, 14, 16 and 20 mm were set symmetrically about the central axis. Monitor unit (MU) of 600 were delivered for each slit field irradiation. A dose-response curve was acquired for 6 MV photon beams for each dose level measurement from 0 to 600 cGy in 50 cGy intervals.

Radiochromic EBT3 film with high spatial resolution, near-tissue equivalence and weak energy dependence has been proven a viable tool for external beam dosimetry. [14–17] All films used in this study were from the same lot number. Each film sheet of 25 × 20 cm^2^ was cut into smaller pieces, 4 × 4 cm^2^ in size for dose-response calibrations and a 10 × 5 cm^2^ for slit field measurements. Film scans were performed at least 24 h after film exposure using an Epson Expression 10000XL document flat-bed scanner (Seiko Epson Corp, Nagano, Japan). Calibration films and measurement films were scanned at the same time to eliminate time-dependent self-developing effect. Each film was scanned at the center of the scanner bed to produce better scanner response uniformity. Film was placed in the landscape orientation with the shorter film side parallel to the scanner detector movement direction. To minimize the lateral dependence artifact effects, a 12 × 12 cm^2^ cardboard template was fitted to the scanner to position films at a reproducible central location. The films were scanned in transmission mode with settings of 72 dpi and 48 bit RGB mode. Images were exported in tagged image file format (TIFF) for analysis. Any manipulation for image processing filters in the scanner operation software were disabled. The red channel data with 16 bit digital information (pixel value, PV) were extracted and processed using the public domain software ImageJ Version 1.43 (National Institute of Health, Bethesda,MD). The net optical densities (netOD) were calculated by subtracting the non-irradiation OD value. The dose response curve was fitted using a netOD-to-dose polynomial function and applied to each measurement film to convert the dose.

After conversion to dose, dose profiles across the films about the central x-axis for each slit field were obtained. The integral dose of each profile was calculated accordingly. The DLGs were derived with the same method used in the ion chamber measurements. Additionally, to eliminate the dose response uncertainty for each film, the background was individually subtracted for each film according to the dose profile at the part of far off from the slit filed to produce the same background tail in the profile for each film.

### Optimal DLG determined with clinical dynamic MLC plans

The DLG values and MLC transmissions were further optimized in TPS while the initial values were based on the ion chamber and film measurements. Twenty-seven IMRT plans and ten RapidArc plans were used in this study, including the plans for the head and neck, lung, breast, esophagus, prostate, endometrium, liver and bone metastasis. The beam energies used in these test plans included all energies analyzed in this study. The γ-index [18] was used for quantifying the agreement between the calculations and measurements with the γ-index criteria of 3% (dose difference) and 3 mm (distance to agreement). A commercially available 2D detector array system (MatriXX, IBA dosimetry, GmbH, Germany) and plastic solid water phantom were used for the planar dose measurements and the comparisons to the TPS calculations [19, 20]. The measurement plane was set at the target center. The DLG value and MLC transmission were then iteratively altered until the optimal values were identified that resulted in the best overall agreement between the calculated and measured dose for the test plans.

## Results

### Cross-field dose width

**Fig. 1:**
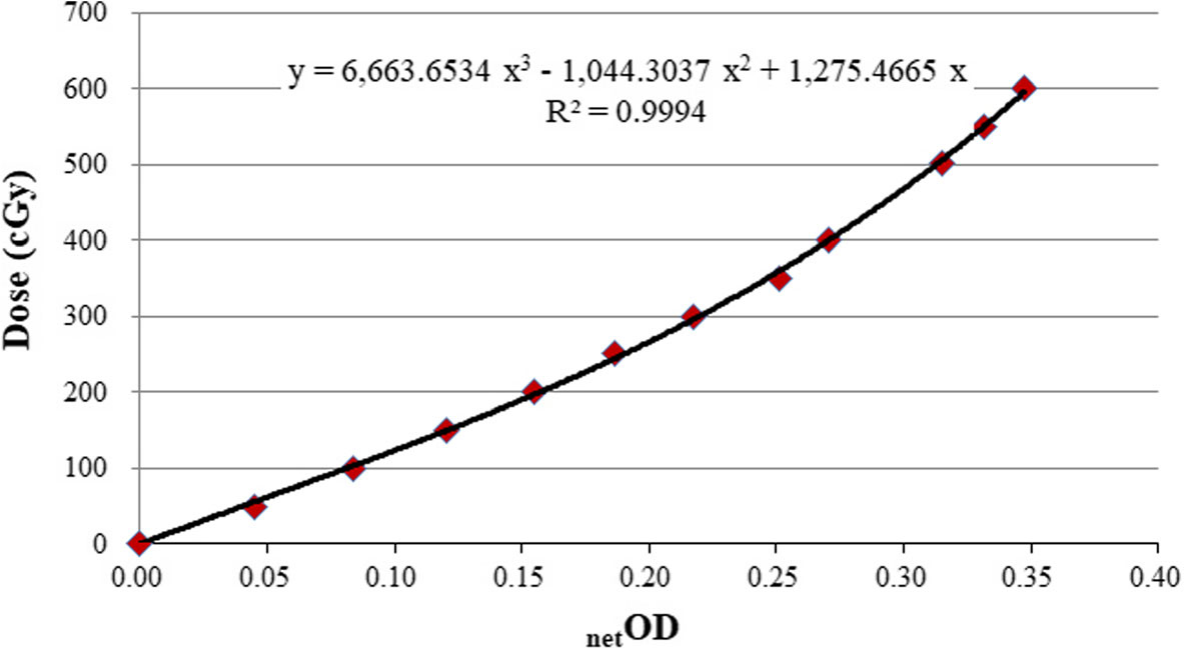
The EBT3 film sensitometric curve

The sensitometric curve of EBT3 film is shown in Fig. 1, and was fitted with a third order polynomial function. Fitting parameters and the agreement of the fit, the coefficient of determination (*R*^*2*^), were also reported. During this study, the *R*^*2*^ values were kept in the 0.9994–0.9997 (0.9996 ± 0.0002) range, which implied good stability of the EBT3 film measurements.

**Fig. 2:**
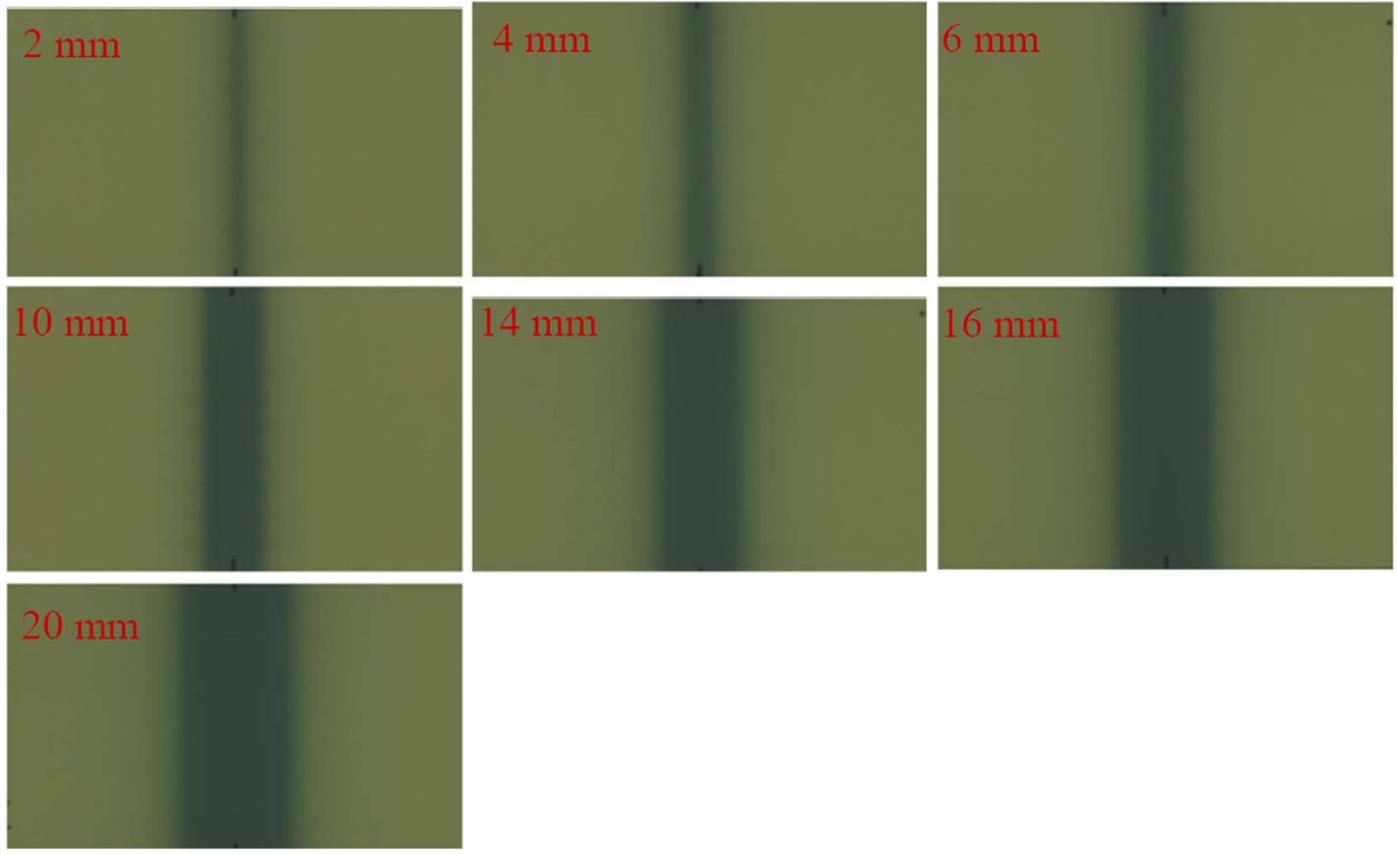
The nominal MLC irradiated film gap sizes of 2, 4, 6, 10, 14, 16 and 20 mm

**Fig. 3:**
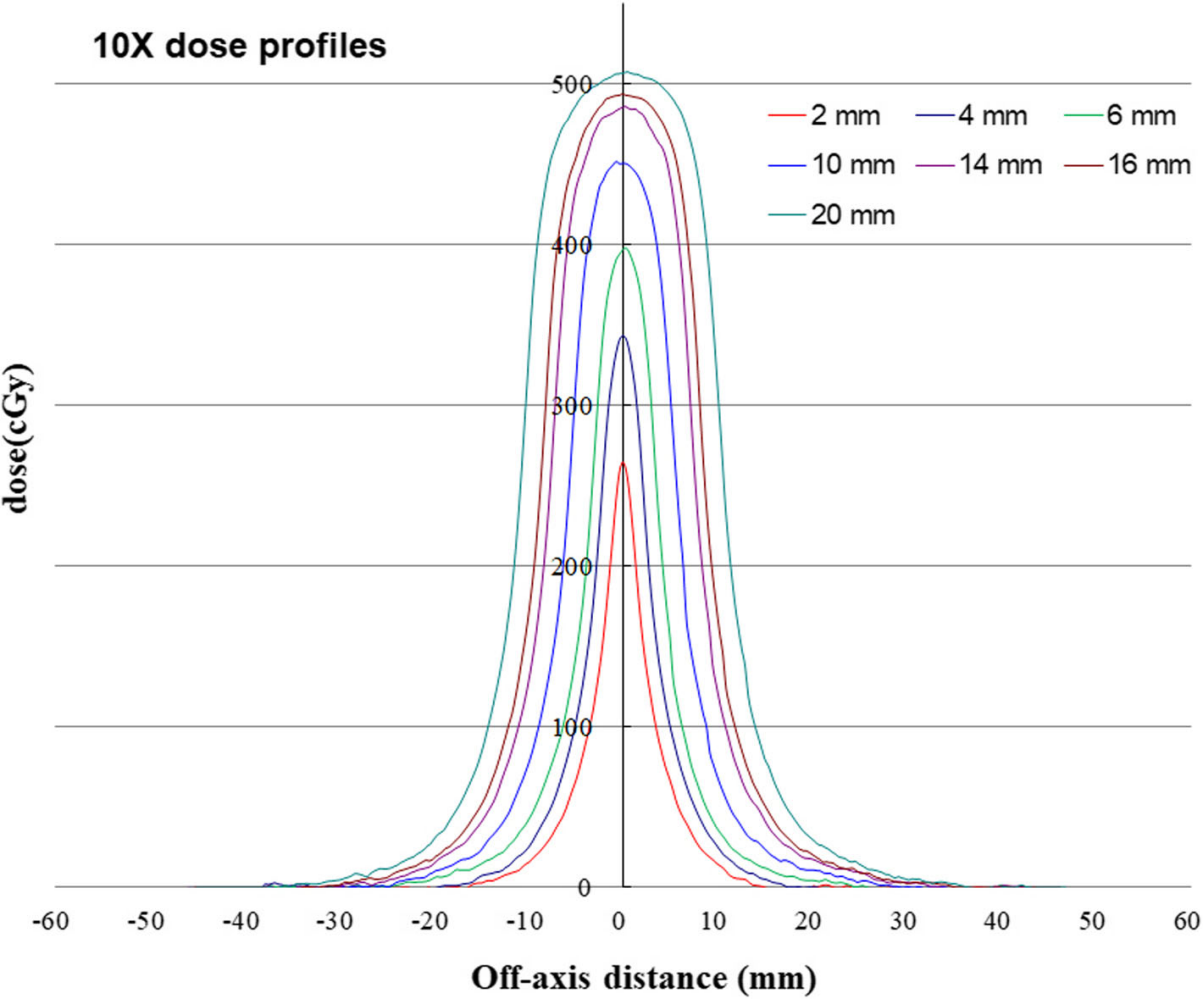
The film measurements of nominal MLC gap dose profiles in sizes of 2, 4, 6, 10, 14, 16 and 20 mm

The irradiated films of the nominal MLC gap sizes of 2, 4, 6, 10, 14, 16 and 20 mm are shown in Fig. 2, and the dose profiles are shown in Fig. 3. As shown in Fig. 3, the dose distribution range is obviously much larger than the nominal gap width. According to the film measurements, the cross-field dose widths (*g*_*D*_) for different nominal gap sizes (*g*_*N*_) of 2, 4, 6, 10, 14, 16 and 20 mm were 30, 40, 50, 60, 70, 80 and 90 mm, respectively. Consequently the *R*_*gT*_ in eq. (1) was calculated with *g*_*N*_ and *g*_*D*_ for different gap size separately.

### DLG values and MLC transmission factors

**Fig. 4:**
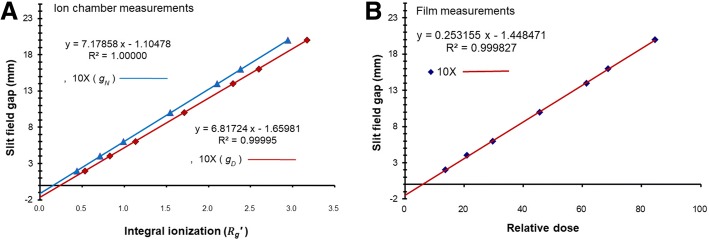
A linear regression analysis for 10 MV measured with the ion chamber (**a**) and the film (**b**). The fitted lines with *Rg*^′^ parameters calculated with *g*_*D*_ and *g*_*N*_ are also shown

A linear regression analysis for 10 MV measured with the ion chamber and the film are shown in Fig. 4. The fitted lines with parameters of *Rg*^′^ calculated with *g*_*D*_ and *g*_*N*_ are also shown, and the difference between the resulting values of DLG is significant.

**Fig. 5:**
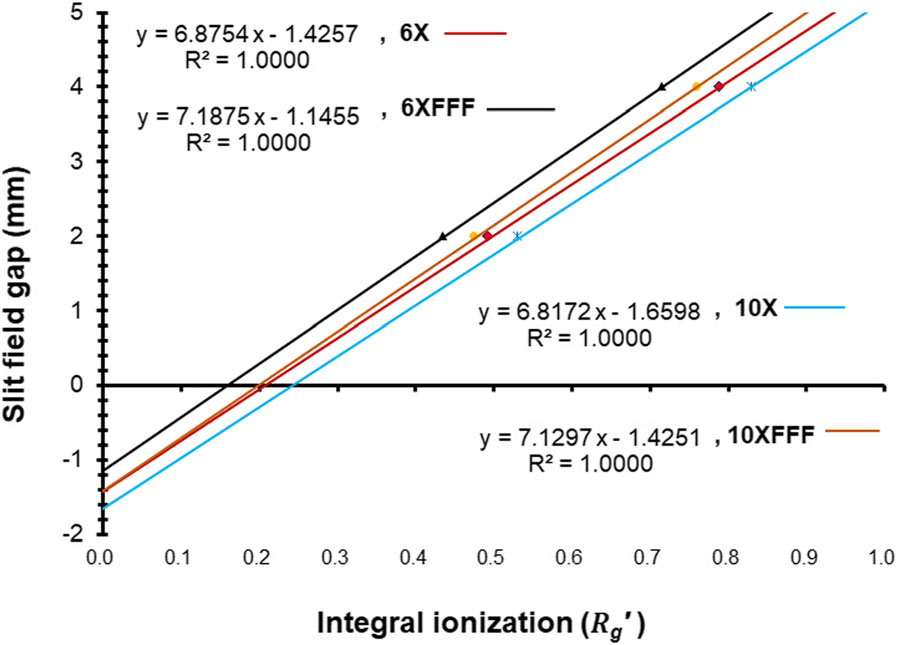
Partial linear regression analysis enlargement for different beam energies measured with the ion chamber with *Rg*^′^ parameters calculated with *g*_*D*_

Figure 5 showed the linear regression for different energies measured with the ion chamber and with *Rg*^′^ parameters calculated with *g*_*D*_. The DLG values, the intercept of the fitted function in Fig. 5 increased with the beam energies.

The optimal DLG values and MLC transmission factors determined with clinical dynamic plans, and the passing rates of the test plans with the γ-index criteria of 3%/3 mm are shown in Table 1. All of the passing rates in this study were higher than 95%. Table 2 shows the measured and optimized DLG values and MLC transmission factors for different beam energies. Similar DLG values were obtained from the ion chamber measurements calculated with *g*_*D*_ and from the film measurements and from the plan optimized values. The difference were within 0.21 mm. Additionally the DLG derived from *g*_*N*_ showed significantly lower values (difference about 0.5 mm) than that derived from *g*_*D*_.

**Table 1:** The averaged Plan QA passing rate (%) with the 3%/3 mm criteria for different delivery techniques having the specified transmission ratio and DLG parameters

	6 MV	6 MV-FFF	10 MV	10 MV-FFF
DLG (mm)	1.40	1.35	1.50	1.45
MLC transmission (%)	1.80	1.60	2.00	1.90
IMRT passing rate (%)	98.63 ± 1.50	98.35 ± 0.29	98.75 ± 1.17	98.07 ± 1.56
Rapidarc passing rate (%)	98.83 ± 0.85	98.35 ± 2.05	99.27 ± 0.61	99.75 ± 0.21

**Table 2:** The measured and optimized DLG and MLC transmission values for different photon energies

		6 MV	6 MV-FFF	10 MV	10 MV-FFF
MLC transmission (%)	Measured	1.74	1.39	2.05	1.70
	Optimized	1.80	1.60	2.00	1.90
DLG (mm)	Measured- ion chamber, *g*_*N*_	0.94	0.75	1.10	0.95
	Measured- ion chamber, *g*_*D*_	1.43	1.15	1.66	1.43
	Measured- Film	1.36	1.26	1.45	1.54
	Optimized	1.40	1.35	1.50	1.45

Table 3 showed the DLG values measured with the ion chamber and film for the depths of 5 and 10 cm. Based on the ion chamber measurements, DLG is insensitive to the depth (differences within 0.07 mm).

**Table 3:** The measured DLG values at depths of 5 and 10 cm for different photon energies

		6 MV	6 MV-FFF	10 MV	10 MV-FFF
DLG (mm)	5 cm- ion chamber, *g*_*D*_	1.43	1.15	1.66	1.43
	10 cm- ion chamber, *g*_*D*_	1.47	1.21	1.68	1.47
	5 cm- Film	1.36	1.26	1.45	1.54
	10 cm- Film	1.35	1.37	1.88	1.65

## Discussion

The DLG and MLC transmission are two important systematic parameters in the dose calculation algorithm of a TPS, especially for a more complex MLC leaf motion. DLG variations are more sensitive to the calculated dose accuracy. However, as the DLG is the parameter that accounts for the additional x-ray transmission through the rounded leaf tips, the DLG value should depend on the beam quality, MLC type and leaf position. Previous studies have shown that the DLG is a spatial variation function and demonstrated that there is no single DLG value for all plan settings [21, 22]. Nevertheless, since the TPS uses a single DLG value to model the opposing leaves offset, it becomes an important step in TPS commissioning for determining an optimal DLG value for each beam energy to calculate doses accurately for the majority of clinically dynamic MLC plans.

For the DLG dosimetric characteristics, the DLG value for different photon energies should be measured directly. Measuring the DLG using the vendor-provided sweeping gap MLC patterns is a convenient and widely used method in clinics. However, TPS with the DLG values measured with this method were found to have significant dose calculation errors during the system investigation. Without a referable DLG value, the iterative process for determining the optimal DLG is time consuming.

This study reevaluated the calculation equation used in the vendor-provided document, and found that this method is not consistent with the method measured with film as described by LoSasso et al. [5]. The ionization reading subtraction contributed by MLC transmission to get the net cumulated ionization should consider the *g*_*D*_. Based on this study, the DLG values derived with parameter of *g*_*D*_ have differences lower than 0.21 mm to the optimal values derived from clinical plans. Using the method proposed in this study, a difference of less than 0.2 mm in DLG value can be obtained, and the value can be used as a starting point to fine tune the optimal DLG more efficiently.

For DLG measurements, Glide-Hurst et al. using a Farmer-type chamber to measure the DLG values for four TrueBeam linacs [13]. The difference in mean DLG values for the 6 MV, 6 MVFFF, 10 MV and 10 MV FFF beams in their report is less than 0.1 mm compared to the values obtained using the methods in our study. Ning Wen et al. using a hybrid approach to optimized the settings of DLG for a TrueBeam linac [11]. The baseline DLG values were measured according to the vendor provided guidelines, and were further optimized in Eclipse. The DLG-measured and the DLG-optimized values were less than 0.06 and 0.23 mm, respectively, compared to the values in our study.

The *g*_*D*_ values were determined using film measurements with fixed slit field irradiations. The energies used in this study were 6 MV, 6 MVFFF, 10 MV and 10MV FFF, and the beam energy dependence on *g*_*D*_ was found to be limited. To simplify the calculation equation, the *g*_*D*_ values for nominal gap sizes of 2, 4, 6, 10, 14, 16 and 20 mm were 30, 40, 50, 60, 70, 80 and 90 mm respectively for all energies used in this study.

The ion chamber provides measurements with low uncertainty, and the measurement with EBT film is a cumbersome process with a relatively higher uncertainty than the ion chamber. However, the method proposed in this study to derive the DLG value must be verified with the method measured with film and with the method by the plan QA. The results in this study encourage us that a simple correction in the calculation equation can lead to DLG values very close to the optimal ones. The difference of less than 0.2 mm in the DLG might be able to keep the PTV dose variation to within 1%.

The DLG characterization has been shown to be insensitive to variations in source to skin distance (SSD), depth of measurement, dose rate, and ionization chamber, while it increases with beam energy [23]. Based on this study in Table 3, the ion chamber measurements also showed the DLG is insensitive to the depth, and increases with beam energy. Note from Table 2 that the DLG values vary linearly with the MLC transmissions. Additionally, a MLC system with less scattering and transmitting radiation should also have a smaller DLG value.

## Conclusions

The DLG values derived from the cross-field dose widths method were consistent with the values derived from the film measurements and from the plan optimal process. A simple and reliable method that determines the DLG for rounded leaf-end MLC systems was established. The DLG value assessment during TPS commissioning can be approached more efficiently and accurately with this method.

## Abbreviations

AAA: Analytical Anisotropic Algorithm
DLG: Dosimetric leaf gap
FFF: Flattening filter free
*g*D: cross-field dose width
*g*N: nominal gap width
IMRT: Intensity-modulated radiotherapy
MLC: Multileaf collimator
MU: Monitor unit
netOD: net optical density
PTV: Planning target volume
RFO: Radiation field offset
*Rg*: initial sweeping gap field reading
*RgT*: average MLC leaf transmission to the gap reading
RT: average MLC leaf transmission
SSD: Source to skin distance
TPS: Treatment planning system
VMAT: Volumetric modulated arc therapy
푅푔′: corrected gap reading
